# Tetra­phenyl­phospho­nium octa­hydro­triborate

**DOI:** 10.1107/S1600536810008263

**Published:** 2010-03-13

**Authors:** Michael A. Beckett, Peter N. Horton, Michael B. Hursthouse, Christian Pszolla

**Affiliations:** a School of Chemistry, University of Wales, Bangor LL57 2UW, Wales; b School of Chemistry, University of Southampton, Highfield, Southampton SO17 1BJ, England

## Abstract

The structure of the title salt, C_24_H_20_P^+^·H_8_B_3_^−^, at 120 (2) K has triclinic (*P*1) symmetry with an unusual *Z* = 5, although there is pseudosymmetry observed with the tetraphenylphosphonium cations exhibiting *I* 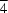 symmetry. One of the anions is disordered over two sets of sites with refined occupancies of 0.478 (11) and 0.522 (11).

## Related literature

For related structures, see: Peters & Nordman (1960 ▶); Mitchell & Welch (1987 ▶); Deiseroth *et al.* (1989 ▶). For synthetic studies of the title compound, see: Amberger & Gut (1968 ▶); Beckett *et al.* (2003 ▶).
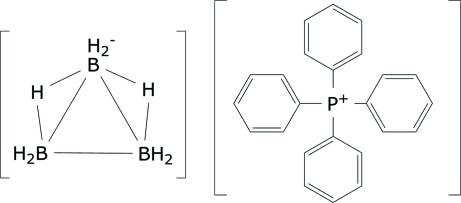


## Experimental

### 

#### Crystal data

C_24_H_20_P^+^·B_3_H_8_^−^
*M* *_r_* = 379.86
Triclinic, 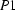

*a* = 7.0314 (5) Å
*b* = 19.820 (2) Å
*c* = 19.832 (2) Å
α = 87.995 (4)°
β = 79.779 (6)°
γ = 79.780 (5)°
*V* = 2676.8 (4) Å^3^
*Z* = 5
Mo *K*α radiation
μ = 0.14 mm^−1^
*T* = 120 K
0.42 × 0.10 × 0.08 mm

#### Data collection

Bruker–Nonius 95mm CCD camera on κ-goniostat diffractometer
Absorption correction: multi-scan (*SADABS*; Bruker, 2003 ▶) *T*_min_ = 0.945, *T*_max_ = 0.989
15011 measured reflections
13369 independent reflections
10251 reflections with *I* > 2σ(*I*)
*R*_int_ = 0.032

#### Refinement

*R*[*F* ^2^ > 2σ(*F* ^2^)] = 0.070
*wR*(*F* ^2^) = 0.191
*S* = 1.02
13369 reflections
1296 parameters
48 restraints
H atoms treated by a mixture of independent and constrained refinement
Δρ_max_ = 0.36 e Å^−3^
Δρ_min_ = −0.52 e Å^−3^
Absolute structure: Flack (1983 ▶), 3867 Friedel pairs
Flack parameter: 0.07 (9)

### 

Data collection: *COLLECT* (Hooft, 1998 ▶); cell refinement: *DENZO* (Otwinowski & Minor, 1997 ▶) and *COLLECT*; data reduction: *DENZO* and *COLLECT*; program(s) used to solve structure: *SHELXS97* (Sheldrick, 2008 ▶); program(s) used to refine structure: *SHELXL97* (Sheldrick, 2008 ▶); molecular graphics: *ORTEP-3 for Windows* (Farrugia, 1997 ▶); software used to prepare material for publication: *WinGX* (Farrugia, 1999 ▶).

## Supplementary Material

Crystal structure: contains datablocks I, global. DOI: 10.1107/S1600536810008263/lh2986sup1.cif

Structure factors: contains datablocks I. DOI: 10.1107/S1600536810008263/lh2986Isup2.hkl

Additional supplementary materials:  crystallographic information; 3D view; checkCIF report

## Figures and Tables

**Fig. 1.**
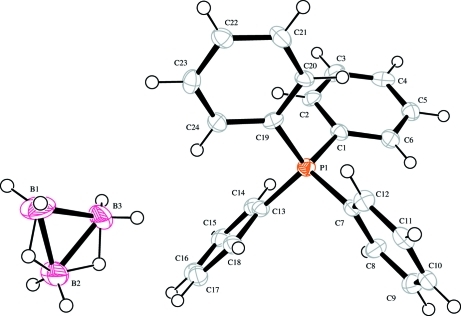
Labelled displacement ellipsoid plot (50% probability) showing one anion and cation (others are similarly labelled).

**Fig. 2.**
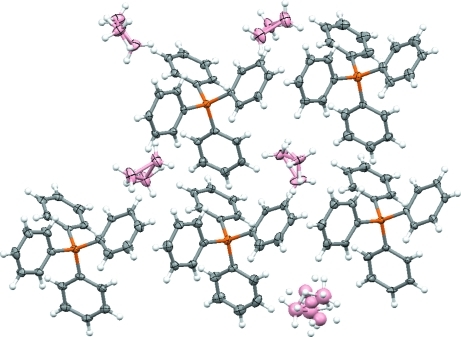
The asymmetric unit of the title compound. The disorder of one of of the anions has been shown.

## supplementary crystallographic information

### Comment

Crystallographically determined structures for salts, [X][B_3_H_8_], which contain the octahydrotriborate(1-)anion, have been restricted to [X] = [(NH_3_)_2_BH_2_] (Peters & Nordman, 1960), [PhCH_2_NMe_3_], (Mitchell & Welch, 1987), and [Cs] (Deiseroth *et al.*, 1989). Preliminary crystallographic data have also been reported for X = [(PPh_3_)_2_N],[NMe_4_], [NEt_4_], [NPr_4_] (Mitchell & Welch, 1987). During the course of our recent synthetic studies (Beckett *et al.*, 2003) we obtained crystals of [Ph_4_P][B_3_H_8_] (1) and these results are reported here. This crystallographic study confirms the ionic nature of 1 with no significant interactions between the ion-pairs. The structure shows considerable pseudo-symmetry with the tetraphenylphosphonium cations exhibiting I4 symmetry. However the smaller octahydrotriborate anions do not, with just one showing two-fold disorder. Hence the requirement to solve in P1.

As has been noted previously in the structures of [(NH_3_)_2_BH_2_][B_3_H_8_] and [PhCH_2_NMe_3_][B_3_H_8_] the B atoms of the octahydrotriborate anions in 1 present themselves as distorted isosceles triangles of approximate *C*_2v_ symmetry.All the B atoms in each [B_3_H_8_]^-^ unit are linked to 2 H atoms in terminal B—H bonds. The other 2 H atoms of the [B_3_H_8_]^-^ unit bridge the B atoms along the shorter sides of the B_3_ triangles.

### Experimental

[Ph_4_P][B_3_H_8_] (1) was prepared from [(C_4_H_9_)_4_N][B_3_H_8_] and [(C_6_H_5_)_4_P]Br in ethanol solution as a colourless crystalline powder by adapting a reported procedure (Amberger & Gut, 1968). A sample suitable for X-ray diffraction was obtained by a slow diffusion of 313-333K pet. ether into a CHCl_3_ solution of 1 at room temperature, m.pt: 518-524K (dec) (lit: 498K, Amberger & Gut, 1968). Calc for C_24_H_28_B_3_P:*C*,77.9%; H, 7.4%. Found C,77.5; H,7.8% .IR/cm^-1^ (nujol mull): 2446; 2392; 2114, 1584; 1138; 1108; 996. NMR(DMSO): d(^1^H/ppm) (250 MHz) 7.87(m; 20H); 0.10 (sept; 8H; *J*_H—B_ = 32.2 Hz); d(^11^B/ppm) (160 MHz): -29.31;d(^13^C/ppm)(125 MHz) 135.3; 134.5; 130.4; 117.7 (d; *J*_C—P_ = 89 Hz); d(^31^P/ppm) (202 MHz): +22.

### Refinement

The initial data collection strategy was for a body-centred tetragonal crystal system (a= 27.593 (2)Å, c= 7.031 (1)Å), with a calculated Z of 10. This data was solvable to a reasonable degree in I4, with an R1=0.124, Z'=1.25, but terrible disorder of the two symmetry inequivalent octahydrotriborate anions. This was not helped by the fact one lay right on the 4-fold rotoinversion axis, for which it is physically impossible to obtain a non-disordered triangle. To help prove likely composition of the compound, this was enough information at the time. To fully prove the structure, further investigations were finally carried out which included lowering the symmetry of the crystal system and space group. It was discovered that when the structure was solved in the reduced triclinic unit cell instead of the original body-centred cell, almost all the disorder disappeared and that without having to use any thermal restraints the displacement parameters were also reasonable. Although the raw data was reintegrated as triclinic to obtain the necessary weak reflections and differing intensities which broke the body-centred tetragonal reflection rules, there was still an overall lack of data since the strategy for the data collection had been based on the tetragonal unit cell (77.6% complete against 99.7% complete out to 0.77Å resolution). This does present a few problems with regard to the overall quality of the structure found. With many fewer equivalent reflections collected, there will be consequential drop-off in the accuracy of the scaling of the data and the absorption correction carried out. This is still enough information for the clear proof of structure, however there is a very slight (but still noticeable) increase in the e.s.d.s of all the bond lengths, angles and the displacement parameters. These still lie within acceptable limits but are all slightly larger than normal for a low temperature crystal structure.

The hydrogen atoms of the phenyl rings were placed at calculated positions using the standard riding model in SHELX for aromatic hydrogen atoms. The terminal (non-bridging) hydrogen atoms of the octahydrotriborate anion was also placed at calculated positions using the standard riding model, this time for methylene hydrogen atoms. The bridging hydrogen atoms were located whenever possible from the difference map. When this was not possible the three boron-boron distances of the triangle were checked and a hydrogen atom was initially placed at the mid-point of the two shorter B-B bonds. No matter how they were located, all the bridging hydrogen atoms were restrained by 3 distance restraints, which were based on average distances for these compounds already found in the Cambridge Structural Database. These distances were 1.16 (6)Å from the two boron atoms it was bridging across and 2.30 (6) Å from the opposite boron atom. These constitute most of the restraints used in the structure.

### Crystal data

**Table:**

C_24_H_20_P^+^·B_3_H_8_^−^	*Z* = 5
*M**_r_* = 379.86	*F*(000) = 1010
Triclinic, *P*1	*D*_x_ = 1.178 Mg m^−^^3^
Hall symbol: P 1	Mo *K*α radiation, λ = 0.71073 Å
*a* = 7.0314 (5) Å	Cell parameters from 3613 reflections
*b* = 19.820 (2) Å	θ = 2.9–27.5°
*c* = 19.832 (2) Å	µ = 0.14 mm^−^^1^
α = 87.995 (4)°	*T* = 120 K
β = 79.779 (6)°	Rod, colourless
γ = 79.780 (5)°	0.42 × 0.10 × 0.08 mm
*V* = 2676.8 (4) Å^3^	

### Data collection

**Table:**

Bruker–Nonius 95mm CCD camera on κ-goniostat diffractometer	13369 independent reflections
Radiation source: Bruker–Nonius FR591 rotating anode	10251 reflections with *I* > 2σ(*I*)
10cm confocal mirrors	*R*_int_ = 0.032
Detector resolution: 9.091 pixels mm^-1^	θ_max_ = 27.5°, θ_min_ = 3.0°
φ and ω scans	*h* = −8→9
Absorption correction: multi-scan (*SADABS*; Bruker, 2003)	*k* = −25→19
*T*_min_ = 0.945, *T*_max_ = 0.989	*l* = −25→25
15011 measured reflections	

### Refinement

**Table:**

Refinement on *F*^2^	Hydrogen site location: inferred from neighbouring sites
Least-squares matrix: full	H atoms treated by a mixture of independent and constrained refinement
*R*[*F*^2^ > 2σ(*F*^2^)] = 0.070	*w* = 1/[σ^2^(*F*_o_^2^) + (0.0656*P*)^2^ + 2.9193*P*] where *P* = (*F*_o_^2^ + 2*F*_c_^2^)/3
*wR*(*F*^2^) = 0.191	(Δ/σ)_max_ < 0.001
*S* = 1.02	Δρ_max_ = 0.36 e Å^−^^3^
13369 reflections	Δρ_min_ = −0.52 e Å^−^^3^
1296 parameters	Extinction correction: *SHELXL97* (Sheldrick, 2008), Fc^*^=kFc[1+0.001xFc^2^λ^3^/sin(2θ)]^-1/4^
48 restraints	Extinction coefficient: 0.0055 (11)
Primary atom site location: structure-invariant direct methods	Absolute structure: Flack (1983), 3867 Friedel pairs
Secondary atom site location: difference Fourier map	Flack parameter: 0.07 (9)

### Special details

**Table:**

Experimental. The data was collected based on a strategy for a body-centred tetragonal crystal system. Only during much later analysis was it noticed that it should be integrated using the primitive crystal system as presented.SADABS was used to perform the Absorption correction,Estimated minimum and maximum transmission: 0.6028 0.7455The given Tmin and Tmax were generated using the SHELX SIZE command
Geometry. All esds (except the esd in the dihedral angle between two l.s. planes) are estimated using the full covariance matrix. The cell esds are taken into account individually in the estimation of esds in distances, angles and torsion angles; correlations between esds in cell parameters are only used when they are defined by crystal symmetry. An approximate (isotropic) treatment of cell esds is used for estimating esds involving l.s. planes.
Refinement. Refinement of *F*^2^ against ALL reflections. The weighted *R*-factor wR and goodness of fit *S* are based on *F*^2^, conventional *R*-factors *R* are based on *F*, with *F* set to zero for negative *F*^2^. The threshold expression of *F*^2^ > σ(*F*^2^) is used only for calculating *R*-factors(gt) etc. and is not relevant to the choice of reflections for refinement. *R*-factors based on *F*^2^ are statistically about twice as large as those based on *F*, and *R*- factors based on ALL data will be even larger.One of the octahydrotriborate anions was disordered over two sites. Due to resulting low expected electron density for each boron atom in this anion, they were left isotropic.For each octahydrotriborate anion, the two bridging hydrogen atoms were located using a combination of either from the difference map and/or based on the boron-boron distances. They were then restrained to suitable distances from the boron atoms (1.16 (6)Å for the two adjacent and 2.30 (6)Å for the one opposite respectively).

### Fractional atomic coordinates and isotropic or equivalent isotropic displacement parameters (Å^2^)

**Table:**

	*x*	*y*	*z*	*U*_iso_*/*U*_eq_	Occ. (<1)
C1	0.9032 (8)	0.7239 (3)	0.6798 (3)	0.0226 (12)	
C2	1.0419 (7)	0.6877 (3)	0.7169 (3)	0.0244 (12)	
H2	1.0808	0.6395	0.7118	0.029*	
C3	1.1218 (8)	0.7234 (3)	0.7612 (3)	0.0307 (14)	
H3	1.2114	0.6994	0.7881	0.037*	
C4	1.0694 (8)	0.7942 (3)	0.7657 (3)	0.0302 (14)	
H4	1.1294	0.8186	0.7940	0.036*	
C5	0.9320 (8)	0.8296 (3)	0.7299 (3)	0.0274 (13)	
H5	0.8943	0.8779	0.7351	0.033*	
C6	0.8481 (8)	0.7950 (3)	0.6861 (3)	0.0265 (13)	
H6	0.7548	0.8194	0.6608	0.032*	
C7	0.6506 (8)	0.7311 (3)	0.5748 (3)	0.0231 (12)	
C8	0.4762 (8)	0.7685 (3)	0.6098 (3)	0.0265 (13)	
H8	0.4436	0.7637	0.6581	0.032*	
C9	0.3498 (8)	0.8123 (3)	0.5751 (3)	0.0312 (14)	
H9	0.2325	0.8382	0.5995	0.037*	
C10	0.3969 (8)	0.8182 (3)	0.5037 (3)	0.0266 (13)	
H10	0.3096	0.8471	0.4792	0.032*	
C11	0.5702 (8)	0.7819 (3)	0.4692 (3)	0.0248 (13)	
H11	0.6019	0.7871	0.4209	0.030*	
C12	0.7007 (8)	0.7376 (3)	0.5031 (2)	0.0246 (12)	
H12	0.8193	0.7126	0.4786	0.029*	
C13	0.6567 (7)	0.6190 (3)	0.6728 (3)	0.0214 (12)	
C14	0.6399 (8)	0.6115 (3)	0.7440 (3)	0.0247 (12)	
H14	0.7101	0.6361	0.7684	0.030*	
C15	0.5220 (8)	0.5685 (3)	0.7785 (3)	0.0276 (13)	
H15	0.5092	0.5640	0.8269	0.033*	
C16	0.4210 (8)	0.5316 (3)	0.7426 (3)	0.0294 (13)	
H16	0.3396	0.5020	0.7665	0.035*	
C17	0.4393 (8)	0.5382 (3)	0.6718 (3)	0.0289 (14)	
H17	0.3729	0.5121	0.6474	0.035*	
C18	0.5529 (8)	0.5822 (3)	0.6371 (3)	0.0251 (12)	
H18	0.5614	0.5878	0.5890	0.030*	
C19	1.0076 (7)	0.6260 (3)	0.5677 (3)	0.0219 (12)	
C20	1.1475 (8)	0.6616 (3)	0.5310 (3)	0.0229 (12)	
H20	1.1331	0.7098	0.5364	0.028*	
C21	1.3069 (8)	0.6271 (3)	0.4868 (3)	0.0306 (14)	
H21	1.3997	0.6516	0.4608	0.037*	
C22	1.3303 (8)	0.5563 (3)	0.4808 (3)	0.0260 (13)	
H22	1.4413	0.5322	0.4514	0.031*	
C23	1.1925 (8)	0.5205 (3)	0.5175 (3)	0.0267 (14)	
H23	1.2096	0.4722	0.5128	0.032*	
C24	1.0298 (8)	0.5549 (3)	0.5610 (3)	0.0235 (13)	
H24	0.9351	0.5305	0.5859	0.028*	
P1	0.8045 (2)	0.67515 (11)	0.62393 (11)	0.0222 (3)	
C31	0.1067 (7)	0.9208 (3)	0.0743 (2)	0.0223 (12)	
C32	0.2452 (7)	0.8814 (3)	0.1087 (3)	0.0250 (12)	
H32	0.2838	0.8336	0.1000	0.030*	
C33	0.3260 (8)	0.9125 (3)	0.1557 (3)	0.0337 (14)	
H33	0.4175	0.8854	0.1799	0.040*	
C34	0.2758 (8)	0.9826 (3)	0.1680 (3)	0.0309 (14)	
H34	0.3344	1.0033	0.1996	0.037*	
C35	0.1388 (8)	1.0218 (3)	0.1336 (3)	0.0331 (14)	
H35	0.1025	1.0696	0.1421	0.040*	
C36	0.0537 (8)	0.9911 (3)	0.0865 (3)	0.0289 (13)	
H36	−0.0397	1.0181	0.0629	0.035*	
C37	−0.1541 (8)	0.9395 (3)	−0.0282 (3)	0.0250 (13)	
C38	−0.1168 (8)	0.9525 (3)	−0.0990 (2)	0.0263 (13)	
H38	−0.0018	0.9294	−0.1273	0.032*	
C39	−0.2519 (8)	1.0000 (3)	−0.1266 (3)	0.0301 (14)	
H39	−0.2277	1.0091	−0.1744	0.036*	
C40	−0.4181 (9)	1.0340 (3)	−0.0877 (3)	0.0370 (16)	
H40	−0.5068	1.0663	−0.1087	0.044*	
C41	−0.4590 (9)	1.0218 (3)	−0.0175 (3)	0.0379 (16)	
H41	−0.5738	1.0457	0.0101	0.046*	
C42	−0.3245 (8)	0.9728 (3)	0.0113 (3)	0.0350 (15)	
H42	−0.3519	0.9624	0.0588	0.042*	
C43	0.2100 (8)	0.8368 (3)	−0.0478 (2)	0.0241 (13)	
C44	0.3387 (8)	0.8768 (3)	−0.0846 (2)	0.0271 (13)	
H44	0.3157	0.9250	−0.0776	0.032*	
C45	0.4987 (8)	0.8466 (3)	−0.1309 (3)	0.0333 (15)	
H45	0.5836	0.8741	−0.1567	0.040*	
C46	0.5364 (8)	0.7755 (3)	−0.1400 (3)	0.0289 (14)	
H46	0.6477	0.7547	−0.1715	0.035*	
C47	0.4122 (8)	0.7354 (3)	−0.1031 (3)	0.0307 (14)	
H47	0.4381	0.6871	−0.1093	0.037*	
C48	0.2506 (8)	0.7654 (3)	−0.0572 (3)	0.0291 (14)	
H48	0.1661	0.7376	−0.0318	0.035*	
C49	−0.1296 (7)	0.8175 (3)	0.0560 (2)	0.0248 (13)	
C50	−0.1527 (8)	0.8078 (3)	0.1273 (2)	0.0243 (12)	
H50	−0.0925	0.8338	0.1541	0.029*	
C51	−0.2624 (8)	0.7609 (3)	0.1582 (3)	0.0281 (13)	
H51	−0.2769	0.7541	0.2063	0.034*	
C52	−0.3525 (9)	0.7233 (3)	0.1194 (3)	0.0361 (14)	
H52	−0.4299	0.6914	0.1413	0.043*	
C53	−0.3301 (9)	0.7319 (3)	0.0487 (3)	0.0411 (16)	
H53	−0.3880	0.7050	0.0222	0.049*	
C54	−0.2239 (8)	0.7795 (3)	0.0177 (3)	0.0334 (14)	
H54	−0.2139	0.7870	−0.0302	0.040*	
P2	0.0078 (2)	0.87843 (9)	0.01328 (8)	0.0231 (4)	
C61	0.4435 (8)	0.5336 (3)	0.1910 (3)	0.0243 (13)	
C62	0.4854 (8)	0.5423 (3)	0.1197 (3)	0.0271 (13)	
H62	0.5991	0.5166	0.0930	0.033*	
C63	0.3558 (8)	0.5897 (3)	0.0893 (3)	0.0281 (14)	
H63	0.3818	0.5964	0.0411	0.034*	
C64	0.1913 (9)	0.6269 (3)	0.1272 (3)	0.0357 (15)	
H64	0.1037	0.6583	0.1050	0.043*	
C65	0.1512 (8)	0.6190 (3)	0.1985 (3)	0.0318 (14)	
H65	0.0396	0.6461	0.2249	0.038*	
C66	0.2763 (8)	0.5713 (3)	0.2299 (3)	0.0327 (15)	
H66	0.2482	0.5643	0.2780	0.039*	
C67	0.4585 (7)	0.4106 (3)	0.2743 (3)	0.0251 (13)	
C68	0.4403 (8)	0.3965 (2)	0.3450 (3)	0.0261 (13)	
H68	0.5071	0.4187	0.3729	0.031*	
C69	0.3234 (8)	0.3498 (3)	0.3731 (3)	0.0302 (14)	
H69	0.3069	0.3411	0.4210	0.036*	
C70	0.2301 (8)	0.3156 (3)	0.3326 (3)	0.0328 (14)	
H70	0.1528	0.2829	0.3525	0.039*	
C71	0.2499 (9)	0.3293 (3)	0.2625 (3)	0.0403 (17)	
H71	0.1871	0.3057	0.2344	0.048*	
C72	0.3599 (8)	0.3767 (3)	0.2342 (3)	0.0335 (15)	
H72	0.3699	0.3868	0.1867	0.040*	
C73	0.7011 (7)	0.5133 (3)	0.2945 (2)	0.0218 (12)	
C74	0.8332 (8)	0.4727 (3)	0.3310 (3)	0.0264 (13)	
H74	0.8649	0.4246	0.3239	0.032*	
C75	0.9168 (8)	0.5029 (3)	0.3772 (3)	0.0253 (13)	
H75	1.0027	0.4752	0.4031	0.030*	
C76	0.8765 (8)	0.5738 (3)	0.3863 (2)	0.0265 (13)	
H76	0.9365	0.5943	0.4177	0.032*	
C77	0.7476 (8)	0.6146 (3)	0.3492 (3)	0.0253 (12)	
H77	0.7212	0.6630	0.3546	0.030*	
C78	0.6585 (8)	0.5837 (3)	0.3044 (3)	0.0258 (13)	
H78	0.5674	0.6111	0.2802	0.031*	
C79	0.8046 (8)	0.4296 (3)	0.1723 (3)	0.0259 (13)	
C80	0.9372 (8)	0.4694 (3)	0.1379 (3)	0.0307 (14)	
H80	0.9194	0.5171	0.1470	0.037*	
C81	1.0939 (9)	0.4386 (3)	0.0908 (3)	0.0404 (17)	
H81	1.1833	0.4656	0.0669	0.049*	
C82	1.1235 (9)	0.3686 (3)	0.0778 (3)	0.0375 (16)	
H82	1.2325	0.3478	0.0453	0.045*	
C83	0.9934 (9)	0.3300 (3)	0.1123 (3)	0.0410 (17)	
H83	1.0139	0.2821	0.1040	0.049*	
C84	0.8332 (8)	0.3595 (3)	0.1589 (3)	0.0311 (15)	
H84	0.7428	0.3323	0.1818	0.037*	
P3	0.6012 (2)	0.47180 (8)	0.23352 (8)	0.0242 (5)	
C91	0.5979 (8)	0.2420 (3)	0.7654 (3)	0.0221 (12)	
C92	0.6287 (8)	0.1712 (3)	0.7556 (3)	0.0294 (14)	
H92	0.5415	0.1444	0.7813	0.035*	
C93	0.7856 (9)	0.1403 (3)	0.7087 (3)	0.0312 (14)	
H93	0.8065	0.0922	0.7017	0.037*	
C94	0.9135 (9)	0.1797 (3)	0.6716 (3)	0.0331 (14)	
H94	1.0233	0.1580	0.6400	0.040*	
C95	0.8829 (9)	0.2505 (3)	0.6800 (3)	0.0387 (16)	
H95	0.9678	0.2773	0.6532	0.046*	
C96	0.7271 (8)	0.2811 (3)	0.7280 (3)	0.0335 (15)	
H96	0.7079	0.3291	0.7356	0.040*	
C97	0.2552 (8)	0.2231 (3)	0.8694 (3)	0.0278 (14)	
C98	0.1591 (8)	0.1887 (3)	0.8289 (3)	0.0300 (14)	
H98	0.1699	0.1979	0.7811	0.036*	
C99	0.0486 (8)	0.1413 (3)	0.8593 (3)	0.0366 (15)	
H99	−0.0156	0.1174	0.8319	0.044*	
C100	0.0293 (8)	0.1278 (3)	0.9296 (3)	0.0323 (14)	
H100	−0.0478	0.0953	0.9502	0.039*	
C101	0.1246 (9)	0.1627 (3)	0.9687 (3)	0.0393 (16)	
H101	0.1114	0.1541	1.0166	0.047*	
C102	0.2391 (8)	0.2098 (3)	0.9394 (3)	0.0350 (15)	
H102	0.3056	0.2328	0.9667	0.042*	
C103	0.4996 (8)	0.3248 (3)	0.8886 (3)	0.0294 (14)	
C104	0.4487 (8)	0.3954 (3)	0.9022 (3)	0.0325 (14)	
H104	0.3555	0.4234	0.8793	0.039*	
C105	0.5354 (8)	0.4240 (3)	0.9492 (3)	0.0352 (15)	
H105	0.5022	0.4718	0.9582	0.042*	
C106	0.6703 (9)	0.3831 (4)	0.9830 (3)	0.0433 (17)	
H106	0.7294	0.4031	1.0150	0.052*	
C107	0.7198 (9)	0.3132 (4)	0.9705 (3)	0.0469 (19)	
H107	0.8104	0.2851	0.9944	0.056*	
C108	0.6352 (8)	0.2847 (3)	0.9226 (3)	0.0393 (17)	
H108	0.6709	0.2370	0.9130	0.047*	
C109	0.2363 (8)	0.3459 (3)	0.7855 (3)	0.0270 (14)	
C110	0.2705 (8)	0.3554 (3)	0.7142 (3)	0.0281 (13)	
H110	0.3844	0.3308	0.6866	0.034*	
C111	0.1355 (8)	0.4013 (3)	0.6847 (3)	0.0255 (13)	
H111	0.1560	0.4077	0.6364	0.031*	
C112	−0.0275 (8)	0.4376 (3)	0.7247 (3)	0.0342 (15)	
H112	−0.1185	0.4690	0.7038	0.041*	
C113	−0.0615 (8)	0.4291 (3)	0.7957 (3)	0.0322 (14)	
H113	−0.1732	0.4552	0.8231	0.039*	
C114	0.0704 (8)	0.3820 (3)	0.8259 (3)	0.0299 (14)	
H114	0.0468	0.3747	0.8740	0.036*	
P4	0.3986 (2)	0.28435 (8)	0.82730 (9)	0.0274 (5)	
C121	0.4158 (8)	0.0247 (3)	0.3564 (2)	0.0211 (12)	
C122	0.4478 (8)	−0.0453 (3)	0.3444 (2)	0.0222 (12)	
H122	0.3602	−0.0731	0.3682	0.027*	
C123	0.6113 (8)	−0.0745 (3)	0.2968 (2)	0.0262 (13)	
H123	0.6350	−0.1223	0.2880	0.031*	
C124	0.7387 (8)	−0.0334 (3)	0.2623 (3)	0.0279 (13)	
H124	0.8487	−0.0535	0.2299	0.034*	
C125	0.7070 (9)	0.0361 (3)	0.2748 (3)	0.0339 (15)	
H125	0.7940	0.0639	0.2508	0.041*	
C126	0.5460 (8)	0.0654 (3)	0.3231 (3)	0.0318 (14)	
H126	0.5256	0.1129	0.3331	0.038*	
C127	0.0506 (7)	0.1278 (2)	0.3767 (3)	0.0206 (12)	
C128	0.0908 (8)	0.1408 (3)	0.3057 (2)	0.0255 (13)	
H128	0.2073	0.1177	0.2782	0.031*	
C129	−0.0432 (9)	0.1880 (3)	0.2767 (3)	0.0330 (15)	
H129	−0.0186	0.1971	0.2289	0.040*	
C130	−0.2115 (8)	0.2217 (3)	0.3169 (3)	0.0329 (15)	
H130	−0.3015	0.2538	0.2963	0.040*	
C131	−0.2518 (8)	0.2094 (3)	0.3873 (3)	0.0300 (13)	
H131	−0.3665	0.2336	0.4148	0.036*	
C132	−0.1211 (8)	0.1611 (3)	0.4165 (3)	0.0261 (13)	
H132	−0.1494	0.1508	0.4639	0.031*	
C133	0.3060 (8)	0.1085 (3)	0.4802 (2)	0.0216 (12)	
C134	0.2572 (8)	0.1795 (3)	0.4895 (3)	0.0257 (12)	
H134	0.1707	0.2067	0.4635	0.031*	
C135	0.3369 (8)	0.2094 (3)	0.5372 (3)	0.0285 (13)	
H135	0.3017	0.2574	0.5449	0.034*	
C136	0.4680 (9)	0.1702 (3)	0.5742 (3)	0.0358 (14)	
H136	0.5253	0.1919	0.6056	0.043*	
C137	0.5153 (8)	0.0999 (3)	0.5655 (3)	0.0330 (14)	
H137	0.5993	0.0731	0.5925	0.040*	
C138	0.4396 (8)	0.0688 (3)	0.5170 (3)	0.0280 (13)	
H138	0.4777	0.0209	0.5088	0.034*	
C139	0.0715 (7)	0.0052 (2)	0.4604 (2)	0.0192 (12)	
C140	−0.0207 (7)	−0.0316 (2)	0.4192 (2)	0.0227 (12)	
H140	−0.0056	−0.0241	0.3711	0.027*	
C141	−0.1323 (8)	−0.0784 (3)	0.4501 (2)	0.0250 (13)	
H141	−0.1885	−0.1049	0.4225	0.030*	
C142	−0.1645 (8)	−0.0878 (3)	0.5209 (3)	0.0265 (13)	
H142	−0.2463	−0.1189	0.5415	0.032*	
C143	−0.0747 (8)	−0.0507 (3)	0.5610 (3)	0.0263 (13)	
H143	−0.0939	−0.0574	0.6093	0.032*	
C144	0.0418 (7)	−0.0045 (3)	0.5313 (2)	0.0233 (12)	
H144	0.1016	0.0205	0.5591	0.028*	
P5	0.21242 (19)	0.06636 (7)	0.41815 (8)	0.0201 (4)	
B1	0.8389 (11)	0.3423 (5)	0.4962 (4)	0.057 (2)	
H1A	0.9233	0.3021	0.5130	0.068*	
H1B	0.8693	0.3509	0.4463	0.068*	
B2	0.5909 (11)	0.3613 (4)	0.5369 (4)	0.048 (2)	
H2A	0.4917	0.3798	0.5082	0.058*	
H2B	0.5456	0.3311	0.5749	0.058*	
B3	0.7572 (11)	0.4137 (4)	0.5505 (4)	0.047 (2)	
H3A	0.7990	0.4111	0.5958	0.057*	
H3B	0.7451	0.4598	0.5291	0.057*	
H5A	0.686 (4)	0.335 (3)	0.485 (2)	0.071*	
H5B	0.588 (4)	0.413 (2)	0.564 (3)	0.071*	
B11	0.1122 (12)	0.7480 (5)	0.3615 (4)	0.053 (2)	
H11A	0.0757	0.7694	0.4074	0.063*	
H11B	0.0744	0.7025	0.3587	0.063*	
B12	0.3317 (11)	0.7625 (4)	0.3101 (4)	0.047 (2)	
H12A	0.4092	0.7247	0.2803	0.057*	
H12B	0.4105	0.7915	0.3290	0.057*	
B13	0.1046 (11)	0.8025 (5)	0.2895 (4)	0.056 (2)	
H13A	0.0631	0.7856	0.2490	0.067*	
H13B	0.0645	0.8524	0.2977	0.067*	
H15A	0.279 (4)	0.730 (3)	0.358 (2)	0.084*	
H15B	0.269 (4)	0.808 (3)	0.276 (3)	0.084*	
B21	0.4241 (12)	0.9362 (4)	0.6960 (4)	0.055 (2)	
H21A	0.4358	0.8902	0.7177	0.066*	
H21B	0.5097	0.9386	0.6510	0.066*	
B22	0.1926 (11)	0.9890 (4)	0.7092 (4)	0.048 (2)	
H22A	0.1563	1.0193	0.6711	0.058*	
H22B	0.0824	0.9709	0.7378	0.058*	
B23	0.3777 (13)	1.0073 (5)	0.7495 (5)	0.064 (3)	
H23A	0.3654	0.9989	0.7994	0.077*	
H23B	0.4392	1.0473	0.7327	0.077*	
H25A	0.276 (5)	0.942 (3)	0.675 (3)	0.096*	
H25B	0.231 (6)	1.0365 (18)	0.736 (3)	0.096*	
B31	0.8983 (13)	0.5483 (5)	0.9568 (5)	0.063 (3)	
H31A	0.7983	0.5654	0.9970	0.076*	
H31B	0.9165	0.4984	0.9488	0.076*	
B32	1.1022 (12)	0.5873 (5)	0.9375 (4)	0.051 (2)	
H32A	1.2293	0.5583	0.9191	0.061*	
H32B	1.1111	0.6253	0.9673	0.061*	
B33	0.9234 (12)	0.6017 (5)	0.8855 (4)	0.056 (2)	
H33A	0.8372	0.6471	0.8879	0.067*	
H33B	0.9556	0.5800	0.8397	0.067*	
H35A	1.069 (5)	0.5322 (19)	0.960 (3)	0.084*	
H35B	1.073 (6)	0.618 (3)	0.886 (2)	0.084*	
B41	0.729 (3)	1.1711 (16)	0.1193 (16)	0.096 (8)*	0.478 (11)
H41A	0.7911	1.1994	0.1463	0.116*	0.478 (11)
H41B	0.8224	1.1375	0.0883	0.116*	0.478 (11)
B42	0.501 (3)	1.1477 (13)	0.1583 (12)	0.093 (8)*	0.478 (11)
H42A	0.4760	1.1021	0.1475	0.111*	0.478 (11)
H42B	0.4447	1.1639	0.2055	0.111*	0.478 (11)
B43	0.510 (3)	1.2096 (11)	0.0919 (11)	0.079 (7)*	0.478 (11)
H43A	0.4891	1.1963	0.0465	0.094*	0.478 (11)
H43B	0.4579	1.2581	0.1045	0.094*	0.478 (11)
B141	0.565 (3)	1.2027 (9)	0.1591 (9)	0.067 (5)*	0.522 (11)
H14A	0.5681	1.1903	0.2078	0.081*	0.522 (11)
H14B	0.6021	1.2476	0.1449	0.081*	0.522 (11)
B142	0.628 (2)	1.1346 (9)	0.0970 (9)	0.065 (5)*	0.522 (11)
H14C	0.6986	1.1445	0.0510	0.078*	0.522 (11)
H14D	0.6646	1.0873	0.1137	0.078*	0.522 (11)
B143	0.386 (2)	1.1751 (9)	0.1202 (9)	0.054 (4)*	0.522 (11)
H14E	0.2940	1.1489	0.1488	0.065*	0.522 (11)
H14F	0.3280	1.2060	0.0861	0.065*	0.522 (11)
H45A	0.670 (6)	1.121 (3)	0.145 (5)	0.081*	0.478 (11)
H45B	0.402 (6)	1.201 (3)	0.145 (3)	0.081*	0.478 (11)
H15G	0.706 (4)	1.170 (7)	0.128 (7)	0.081*	0.522 (11)
H15H	0.491 (6)	1.150 (4)	0.069 (2)	0.081*	0.522 (11)

### Atomic displacement parameters (Å^2^)

**Table:**

	*U*^11^	*U*^22^	*U*^33^	*U*^12^	*U*^13^	*U*^23^
C1	0.021 (3)	0.024 (3)	0.023 (3)	−0.007 (2)	0.001 (2)	−0.004 (2)
C2	0.018 (3)	0.025 (3)	0.028 (3)	0.002 (2)	−0.004 (2)	−0.011 (2)
C3	0.023 (3)	0.032 (3)	0.036 (3)	0.002 (3)	−0.008 (3)	−0.007 (3)
C4	0.019 (3)	0.045 (4)	0.027 (3)	−0.009 (3)	−0.001 (3)	−0.005 (3)
C5	0.026 (3)	0.025 (3)	0.029 (3)	−0.006 (2)	0.004 (3)	−0.003 (2)
C6	0.026 (3)	0.027 (3)	0.025 (3)	−0.007 (2)	0.001 (3)	0.003 (2)
C7	0.022 (3)	0.021 (3)	0.030 (3)	−0.010 (2)	−0.009 (3)	0.007 (2)
C8	0.022 (3)	0.030 (3)	0.025 (3)	0.000 (3)	−0.002 (3)	0.004 (2)
C9	0.025 (3)	0.032 (3)	0.034 (3)	−0.003 (3)	−0.003 (3)	0.006 (3)
C10	0.027 (3)	0.023 (3)	0.033 (3)	−0.010 (3)	−0.008 (3)	0.004 (2)
C11	0.032 (4)	0.029 (3)	0.018 (2)	−0.015 (3)	−0.007 (3)	0.003 (2)
C12	0.026 (3)	0.026 (3)	0.021 (2)	−0.003 (2)	−0.002 (2)	−0.003 (2)
C13	0.018 (3)	0.023 (3)	0.022 (2)	−0.003 (2)	−0.003 (2)	0.004 (2)
C14	0.024 (3)	0.024 (3)	0.025 (3)	0.000 (2)	−0.003 (2)	−0.005 (2)
C15	0.021 (3)	0.027 (3)	0.028 (3)	0.004 (2)	0.005 (3)	−0.003 (2)
C16	0.022 (3)	0.026 (3)	0.037 (3)	−0.001 (2)	−0.001 (3)	0.000 (2)
C17	0.033 (4)	0.026 (3)	0.032 (3)	−0.007 (2)	−0.015 (3)	0.004 (2)
C18	0.025 (3)	0.027 (3)	0.025 (3)	−0.005 (2)	−0.007 (3)	0.001 (2)
C19	0.017 (3)	0.027 (3)	0.019 (2)	0.001 (2)	−0.002 (2)	−0.004 (2)
C20	0.017 (3)	0.023 (3)	0.027 (3)	−0.006 (2)	0.004 (3)	−0.006 (2)
C21	0.031 (4)	0.029 (3)	0.035 (3)	−0.015 (3)	−0.003 (3)	0.000 (3)
C22	0.025 (3)	0.030 (3)	0.027 (3)	−0.009 (2)	−0.010 (3)	0.002 (2)
C23	0.035 (4)	0.020 (3)	0.026 (3)	−0.001 (3)	−0.011 (3)	−0.003 (2)
C24	0.025 (3)	0.025 (3)	0.022 (3)	−0.007 (2)	−0.005 (3)	−0.002 (2)
P1	0.0217 (8)	0.0226 (6)	0.0227 (6)	−0.0036 (5)	−0.0052 (6)	−0.0003 (5)
C31	0.014 (3)	0.037 (3)	0.013 (2)	−0.006 (2)	0.004 (2)	−0.005 (2)
C32	0.019 (3)	0.026 (3)	0.028 (3)	−0.001 (2)	0.001 (3)	−0.005 (2)
C33	0.023 (3)	0.046 (4)	0.031 (3)	−0.003 (3)	−0.005 (3)	0.003 (3)
C34	0.022 (3)	0.044 (4)	0.026 (3)	−0.013 (3)	0.003 (3)	−0.002 (3)
C35	0.025 (4)	0.039 (3)	0.029 (3)	−0.009 (3)	0.016 (3)	0.000 (3)
C36	0.027 (3)	0.023 (3)	0.034 (3)	−0.006 (2)	0.003 (3)	0.007 (2)
C37	0.019 (3)	0.030 (3)	0.024 (3)	−0.003 (2)	−0.001 (3)	0.001 (2)
C38	0.022 (3)	0.032 (3)	0.023 (3)	−0.001 (2)	−0.002 (3)	−0.001 (2)
C39	0.028 (4)	0.032 (3)	0.028 (3)	0.000 (3)	−0.005 (3)	0.003 (3)
C40	0.033 (4)	0.038 (4)	0.041 (3)	−0.004 (3)	−0.015 (3)	0.016 (3)
C41	0.023 (4)	0.047 (4)	0.038 (3)	0.001 (3)	0.001 (3)	0.015 (3)
C42	0.029 (4)	0.047 (4)	0.026 (3)	−0.007 (3)	0.000 (3)	0.009 (3)
C43	0.019 (3)	0.035 (3)	0.017 (3)	−0.001 (3)	−0.001 (2)	−0.003 (2)
C44	0.026 (4)	0.032 (3)	0.022 (3)	−0.006 (3)	0.000 (3)	−0.001 (2)
C45	0.027 (4)	0.044 (4)	0.030 (3)	−0.011 (3)	−0.002 (3)	−0.003 (3)
C46	0.022 (3)	0.037 (3)	0.025 (3)	0.001 (3)	−0.003 (3)	−0.009 (3)
C47	0.029 (4)	0.035 (3)	0.027 (3)	−0.003 (3)	−0.005 (3)	−0.003 (3)
C48	0.029 (4)	0.037 (3)	0.020 (3)	−0.005 (3)	−0.002 (3)	0.004 (2)
C49	0.018 (3)	0.036 (3)	0.021 (3)	−0.007 (2)	−0.004 (2)	0.003 (2)
C50	0.025 (3)	0.026 (3)	0.018 (2)	−0.001 (2)	0.004 (2)	−0.002 (2)
C51	0.029 (3)	0.034 (3)	0.020 (3)	−0.007 (3)	0.001 (3)	0.013 (2)
C52	0.024 (3)	0.041 (3)	0.043 (3)	−0.011 (3)	−0.002 (3)	0.010 (3)
C53	0.033 (4)	0.048 (4)	0.048 (4)	−0.019 (3)	−0.014 (3)	0.011 (3)
C54	0.029 (4)	0.042 (4)	0.032 (3)	−0.014 (3)	−0.006 (3)	0.004 (3)
P2	0.0217 (9)	0.0299 (10)	0.0171 (9)	−0.0043 (7)	−0.0029 (8)	0.0016 (8)
C61	0.023 (3)	0.023 (3)	0.027 (3)	−0.004 (2)	−0.004 (3)	−0.004 (2)
C62	0.027 (3)	0.029 (3)	0.026 (3)	−0.006 (2)	−0.008 (3)	−0.005 (2)
C63	0.029 (4)	0.030 (3)	0.028 (3)	−0.007 (3)	−0.009 (3)	0.003 (2)
C64	0.032 (4)	0.038 (3)	0.040 (3)	−0.007 (3)	−0.016 (3)	0.004 (3)
C65	0.022 (3)	0.036 (3)	0.032 (3)	0.002 (3)	0.003 (3)	−0.001 (3)
C66	0.029 (4)	0.041 (4)	0.027 (3)	−0.003 (3)	−0.004 (3)	0.000 (3)
C67	0.020 (3)	0.017 (3)	0.040 (3)	−0.002 (2)	−0.012 (3)	−0.002 (2)
C68	0.028 (3)	0.012 (2)	0.040 (3)	−0.007 (2)	−0.005 (3)	−0.006 (2)
C69	0.032 (4)	0.019 (3)	0.037 (3)	0.001 (2)	−0.007 (3)	−0.002 (2)
C70	0.023 (3)	0.020 (3)	0.057 (4)	−0.010 (2)	−0.007 (3)	0.007 (3)
C71	0.041 (4)	0.028 (3)	0.062 (4)	−0.011 (3)	−0.032 (4)	0.003 (3)
C72	0.028 (4)	0.033 (3)	0.043 (3)	−0.008 (3)	−0.013 (3)	0.003 (3)
C73	0.021 (3)	0.024 (3)	0.021 (3)	−0.007 (2)	−0.001 (2)	−0.004 (2)
C74	0.025 (3)	0.017 (3)	0.038 (3)	−0.006 (2)	−0.005 (3)	−0.003 (2)
C75	0.023 (3)	0.022 (3)	0.031 (3)	−0.003 (2)	−0.007 (3)	−0.003 (2)
C76	0.026 (3)	0.032 (3)	0.022 (3)	−0.007 (3)	−0.003 (3)	−0.001 (2)
C77	0.024 (3)	0.019 (3)	0.031 (3)	−0.004 (2)	−0.002 (3)	0.000 (2)
C78	0.026 (3)	0.026 (3)	0.027 (3)	−0.007 (2)	−0.005 (3)	0.003 (2)
C79	0.018 (3)	0.028 (3)	0.032 (3)	−0.002 (2)	−0.004 (3)	−0.006 (2)
C80	0.014 (3)	0.039 (3)	0.040 (3)	−0.007 (3)	−0.003 (3)	−0.010 (3)
C81	0.029 (4)	0.051 (4)	0.045 (4)	−0.010 (3)	−0.014 (3)	−0.008 (3)
C82	0.027 (4)	0.047 (4)	0.039 (3)	−0.001 (3)	−0.015 (3)	−0.007 (3)
C83	0.038 (4)	0.030 (3)	0.057 (4)	0.002 (3)	−0.023 (4)	−0.007 (3)
C84	0.029 (4)	0.031 (3)	0.037 (3)	−0.004 (3)	−0.016 (3)	−0.001 (3)
P3	0.0221 (9)	0.0220 (9)	0.0302 (11)	−0.0046 (7)	−0.0084 (9)	−0.0002 (9)
C91	0.018 (3)	0.019 (3)	0.028 (3)	−0.003 (2)	−0.002 (2)	0.001 (2)
C92	0.023 (3)	0.023 (3)	0.041 (3)	−0.001 (3)	−0.008 (3)	0.011 (3)
C93	0.030 (4)	0.027 (3)	0.034 (3)	0.003 (3)	−0.005 (3)	−0.005 (3)
C94	0.026 (3)	0.037 (3)	0.035 (3)	−0.003 (3)	−0.008 (3)	0.004 (3)
C95	0.028 (4)	0.045 (4)	0.043 (3)	−0.015 (3)	0.002 (3)	0.000 (3)
C96	0.023 (4)	0.025 (3)	0.052 (4)	−0.006 (3)	−0.006 (3)	0.002 (3)
C97	0.020 (3)	0.028 (3)	0.037 (3)	−0.007 (2)	−0.010 (3)	0.006 (3)
C98	0.017 (3)	0.038 (3)	0.034 (3)	−0.002 (3)	−0.005 (3)	0.008 (3)
C99	0.020 (3)	0.042 (4)	0.049 (4)	−0.005 (3)	−0.010 (3)	0.010 (3)
C100	0.019 (3)	0.035 (3)	0.039 (3)	0.000 (3)	−0.001 (3)	0.012 (3)
C101	0.031 (4)	0.047 (4)	0.039 (3)	−0.001 (3)	−0.007 (3)	−0.002 (3)
C102	0.027 (4)	0.037 (4)	0.040 (3)	0.002 (3)	−0.011 (3)	−0.002 (3)
C103	0.022 (3)	0.036 (3)	0.031 (3)	−0.003 (3)	−0.008 (3)	−0.006 (3)
C104	0.028 (3)	0.045 (4)	0.026 (3)	−0.009 (3)	−0.009 (3)	0.003 (3)
C105	0.029 (4)	0.040 (4)	0.035 (3)	−0.006 (3)	0.000 (3)	−0.003 (3)
C106	0.026 (4)	0.069 (5)	0.037 (3)	−0.008 (3)	−0.006 (3)	−0.012 (3)
C107	0.030 (4)	0.059 (5)	0.049 (4)	0.015 (3)	−0.020 (3)	−0.013 (4)
C108	0.023 (3)	0.048 (4)	0.046 (4)	0.005 (3)	−0.012 (3)	−0.016 (3)
C109	0.023 (3)	0.018 (3)	0.041 (3)	−0.005 (2)	−0.009 (3)	0.003 (3)
C110	0.027 (3)	0.026 (3)	0.032 (3)	−0.002 (2)	−0.010 (3)	0.000 (2)
C111	0.022 (3)	0.033 (3)	0.021 (3)	−0.002 (3)	−0.004 (3)	−0.005 (2)
C112	0.031 (4)	0.027 (3)	0.049 (4)	−0.005 (3)	−0.017 (3)	0.006 (3)
C113	0.021 (3)	0.027 (3)	0.045 (3)	0.002 (2)	−0.004 (3)	−0.003 (3)
C114	0.024 (4)	0.033 (3)	0.029 (3)	0.002 (3)	−0.002 (3)	0.001 (3)
P4	0.0221 (9)	0.0286 (12)	0.0325 (11)	−0.0022 (9)	−0.0097 (8)	0.0012 (10)
C121	0.025 (3)	0.024 (3)	0.016 (2)	−0.009 (2)	−0.002 (2)	−0.005 (2)
C122	0.027 (3)	0.022 (3)	0.016 (2)	−0.006 (2)	0.002 (3)	−0.002 (2)
C123	0.033 (4)	0.029 (3)	0.016 (2)	−0.006 (3)	0.000 (3)	−0.006 (2)
C124	0.020 (3)	0.039 (3)	0.022 (3)	−0.006 (3)	0.007 (3)	−0.014 (2)
C125	0.034 (4)	0.034 (3)	0.031 (3)	−0.014 (3)	0.010 (3)	−0.004 (3)
C126	0.031 (4)	0.027 (3)	0.038 (3)	−0.011 (3)	0.002 (3)	−0.012 (3)
C127	0.019 (3)	0.018 (3)	0.027 (3)	−0.009 (2)	−0.003 (3)	0.001 (2)
C128	0.033 (3)	0.026 (3)	0.019 (2)	−0.010 (2)	−0.001 (3)	−0.006 (2)
C129	0.041 (4)	0.027 (3)	0.037 (3)	−0.016 (3)	−0.017 (3)	0.003 (3)
C130	0.026 (4)	0.025 (3)	0.049 (4)	−0.006 (3)	−0.008 (3)	−0.002 (3)
C131	0.024 (3)	0.029 (3)	0.038 (3)	−0.009 (3)	−0.001 (3)	−0.002 (3)
C132	0.016 (3)	0.030 (3)	0.031 (3)	−0.009 (3)	0.003 (3)	−0.001 (2)
C133	0.024 (3)	0.021 (3)	0.019 (2)	−0.007 (2)	0.003 (2)	−0.006 (2)
C134	0.035 (4)	0.019 (3)	0.026 (3)	−0.013 (2)	−0.002 (3)	−0.003 (2)
C135	0.027 (3)	0.029 (3)	0.028 (3)	−0.007 (3)	0.002 (3)	−0.011 (2)
C136	0.033 (4)	0.042 (4)	0.035 (3)	−0.015 (3)	−0.005 (3)	−0.011 (3)
C137	0.030 (3)	0.039 (3)	0.032 (3)	−0.003 (3)	−0.011 (3)	−0.008 (3)
C138	0.025 (3)	0.028 (3)	0.029 (3)	−0.001 (2)	−0.002 (3)	−0.013 (2)
C139	0.018 (3)	0.019 (3)	0.020 (2)	−0.006 (2)	0.001 (2)	0.003 (2)
C140	0.024 (3)	0.023 (3)	0.021 (3)	−0.006 (2)	−0.001 (2)	0.000 (2)
C141	0.027 (3)	0.028 (3)	0.022 (3)	−0.009 (2)	−0.004 (3)	0.005 (2)
C142	0.027 (3)	0.022 (3)	0.032 (3)	−0.010 (2)	−0.004 (3)	0.005 (2)
C143	0.029 (3)	0.026 (3)	0.023 (3)	−0.009 (2)	0.001 (3)	0.005 (2)
C144	0.026 (3)	0.020 (3)	0.022 (3)	−0.002 (2)	−0.003 (2)	−0.002 (2)
P5	0.0209 (9)	0.0193 (9)	0.0203 (9)	−0.0053 (8)	−0.0017 (7)	−0.0041 (8)
B1	0.027 (5)	0.077 (6)	0.061 (5)	−0.002 (4)	0.006 (4)	−0.028 (5)
B2	0.037 (5)	0.052 (5)	0.058 (5)	−0.008 (4)	−0.005 (4)	−0.035 (4)
B3	0.038 (5)	0.038 (4)	0.068 (5)	−0.013 (4)	−0.007 (4)	−0.023 (4)
B11	0.045 (5)	0.058 (5)	0.051 (4)	−0.007 (4)	−0.003 (4)	0.023 (4)
B12	0.033 (4)	0.059 (5)	0.056 (5)	−0.019 (4)	−0.020 (4)	0.027 (4)
B13	0.038 (5)	0.066 (6)	0.070 (5)	−0.017 (4)	−0.027 (4)	0.034 (5)
B21	0.039 (5)	0.058 (6)	0.059 (5)	0.013 (4)	−0.001 (4)	−0.011 (4)
B22	0.038 (5)	0.049 (5)	0.060 (5)	−0.003 (4)	−0.015 (4)	−0.019 (4)
B23	0.051 (6)	0.078 (7)	0.067 (6)	−0.017 (5)	−0.010 (5)	−0.022 (5)
B31	0.050 (6)	0.063 (6)	0.077 (6)	−0.015 (5)	−0.012 (5)	0.031 (5)
B32	0.043 (5)	0.059 (5)	0.054 (5)	−0.010 (4)	−0.018 (4)	0.022 (4)
B33	0.041 (5)	0.072 (6)	0.059 (5)	−0.009 (4)	−0.028 (4)	0.027 (4)

### Geometric parameters (Å, °)

**Table:**

C1—C6	1.396 (7)	C99—H99	0.9500
C1—C2	1.406 (6)	C100—C101	1.383 (8)
C1—P1	1.797 (5)	C100—H100	0.9500
C2—C3	1.396 (7)	C101—C102	1.387 (8)
C2—H2	0.9500	C101—H101	0.9500
C3—C4	1.387 (7)	C102—H102	0.9500
C3—H3	0.9500	C103—C108	1.384 (7)
C4—C5	1.378 (7)	C103—C104	1.405 (8)
C4—H4	0.9500	C103—P4	1.790 (5)
C5—C6	1.393 (7)	C104—C105	1.386 (7)
C5—H5	0.9500	C104—H104	0.9500
C6—H6	0.9500	C105—C106	1.387 (8)
C7—C8	1.394 (7)	C105—H105	0.9500
C7—C12	1.410 (7)	C106—C107	1.388 (9)
C7—P1	1.795 (5)	C106—H106	0.9500
C8—C9	1.386 (6)	C107—C108	1.391 (7)
C8—H8	0.9500	C107—H107	0.9500
C9—C10	1.402 (7)	C108—H108	0.9500
C9—H9	0.9500	C109—C114	1.388 (8)
C10—C11	1.378 (7)	C109—C110	1.403 (7)
C10—H10	0.9500	C109—P4	1.802 (5)
C11—C12	1.400 (6)	C110—C111	1.388 (6)
C11—H11	0.9500	C110—H110	0.9500
C12—H12	0.9500	C111—C112	1.373 (8)
C13—C14	1.400 (7)	C111—H111	0.9500
C13—C18	1.406 (6)	C112—C113	1.395 (8)
C13—P1	1.799 (5)	C112—H112	0.9500
C14—C15	1.375 (7)	C113—C114	1.393 (7)
C14—H14	0.9500	C113—H113	0.9500
C15—C16	1.396 (7)	C114—H114	0.9500
C15—H15	0.9500	C121—C122	1.388 (7)
C16—C17	1.390 (7)	C121—C126	1.391 (7)
C16—H16	0.9500	C121—P5	1.804 (5)
C17—C18	1.372 (7)	C122—C123	1.402 (7)
C17—H17	0.9500	C122—H122	0.9500
C18—H18	0.9500	C123—C124	1.391 (8)
C19—C24	1.398 (7)	C123—H123	0.9500
C19—C20	1.398 (7)	C124—C125	1.380 (8)
C19—P1	1.803 (5)	C124—H124	0.9500
C20—C21	1.383 (8)	C125—C126	1.399 (8)
C20—H20	0.9500	C125—H125	0.9500
C21—C22	1.391 (7)	C126—H126	0.9500
C21—H21	0.9500	C127—C132	1.389 (7)
C22—C23	1.388 (7)	C127—C128	1.410 (7)
C22—H22	0.9500	C127—P5	1.794 (5)
C23—C24	1.390 (7)	C128—C129	1.392 (7)
C23—H23	0.9500	C128—H128	0.9500
C24—H24	0.9500	C129—C130	1.380 (8)
C31—C36	1.395 (7)	C129—H129	0.9500
C31—C32	1.397 (6)	C130—C131	1.396 (8)
C31—P2	1.798 (5)	C130—H130	0.9500
C32—C33	1.387 (7)	C131—C132	1.391 (7)
C32—H32	0.9500	C131—H131	0.9500
C33—C34	1.389 (8)	C132—H132	0.9500
C33—H33	0.9500	C133—C134	1.398 (7)
C34—C35	1.388 (7)	C133—C138	1.407 (6)
C34—H34	0.9500	C133—P5	1.788 (5)
C35—C36	1.404 (7)	C134—C135	1.380 (6)
C35—H35	0.9500	C134—H134	0.9500
C36—H36	0.9500	C135—C136	1.392 (7)
C37—C42	1.381 (8)	C135—H135	0.9500
C37—C38	1.407 (7)	C136—C137	1.383 (8)
C37—P2	1.792 (5)	C136—H136	0.9500
C38—C39	1.384 (7)	C137—C138	1.388 (7)
C38—H38	0.9500	C137—H137	0.9500
C39—C40	1.360 (8)	C138—H138	0.9500
C39—H39	0.9500	C139—C144	1.397 (6)
C40—C41	1.393 (8)	C139—C140	1.420 (6)
C40—H40	0.9500	C139—P5	1.792 (5)
C41—C42	1.412 (7)	C140—C141	1.378 (7)
C41—H41	0.9500	C140—H140	0.9500
C42—H42	0.9500	C141—C142	1.393 (7)
C43—C44	1.401 (7)	C141—H141	0.9500
C43—C48	1.405 (7)	C142—C143	1.399 (7)
C43—P2	1.793 (5)	C142—H142	0.9500
C44—C45	1.379 (8)	C143—C144	1.384 (7)
C44—H44	0.9500	C143—H143	0.9500
C45—C46	1.398 (8)	C144—H144	0.9500
C45—H45	0.9500	B1—B3	1.759 (10)
C46—C47	1.382 (8)	B1—B2	1.767 (10)
C46—H46	0.9500	B1—H1A	0.9900
C47—C48	1.379 (8)	B1—H1B	0.9900
C47—H47	0.9500	B1—H5A	1.17 (3)
C48—H48	0.9500	B2—B3	1.755 (9)
C49—C54	1.405 (7)	B2—H2A	0.9900
C49—C50	1.405 (6)	B2—H2B	0.9900
C49—P2	1.778 (6)	B2—H5A	1.21 (3)
C50—C51	1.372 (7)	B2—H5B	1.18 (3)
C50—H50	0.9500	B3—H3A	0.9900
C51—C52	1.390 (7)	B3—H3B	0.9900
C51—H51	0.9500	B3—H5B	1.17 (3)
C52—C53	1.390 (8)	B11—B12	1.756 (11)
C52—H52	0.9500	B11—B13	1.763 (10)
C53—C54	1.368 (8)	B11—H11A	0.9900
C53—H53	0.9500	B11—H11B	0.9900
C54—H54	0.9500	B11—H15A	1.16 (3)
C61—C66	1.393 (8)	B12—B13	1.762 (9)
C61—C62	1.405 (7)	B12—H12A	0.9900
C61—P3	1.794 (5)	B12—H12B	0.9900
C62—C63	1.387 (6)	B12—H15A	1.18 (3)
C62—H62	0.9500	B12—H15B	1.17 (3)
C63—C64	1.370 (8)	B13—H13A	0.9900
C63—H63	0.9500	B13—H13B	0.9900
C64—C65	1.400 (8)	B13—H15B	1.16 (3)
C64—H64	0.9500	B21—B23	1.742 (11)
C65—C66	1.384 (7)	B21—B22	1.755 (10)
C65—H65	0.9500	B21—H21A	0.9900
C66—H66	0.9500	B21—H21B	0.9900
C67—C72	1.401 (7)	B21—H25A	1.17 (3)
C67—C68	1.407 (7)	B22—B23	1.737 (10)
C67—P3	1.791 (6)	B22—H22A	0.9900
C68—C69	1.383 (7)	B22—H22B	0.9900
C68—H68	0.9500	B22—H25A	1.17 (3)
C69—C70	1.385 (7)	B22—H25B	1.19 (3)
C69—H69	0.9500	B23—H23A	0.9900
C70—C71	1.395 (8)	B23—H23B	0.9900
C70—H70	0.9500	B23—H25B	1.16 (3)
C71—C72	1.364 (8)	B31—B32	1.726 (11)
C71—H71	0.9500	B31—B33	1.738 (11)
C72—H72	0.9500	B31—H31A	0.9900
C73—C78	1.389 (7)	B31—H31B	0.9900
C73—C74	1.402 (6)	B31—H35A	1.20 (3)
C73—P3	1.790 (5)	B32—B33	1.743 (9)
C74—C75	1.378 (6)	B32—H32A	0.9900
C74—H74	0.9500	B32—H32B	0.9900
C75—C76	1.395 (7)	B32—H35A	1.21 (3)
C75—H75	0.9500	B32—H35B	1.20 (3)
C76—C77	1.398 (6)	B33—H33A	0.9900
C76—H76	0.9500	B33—H33B	0.9900
C77—C78	1.389 (7)	B33—H35B	1.15 (3)
C77—H77	0.9500	B41—B43	1.760 (19)
C78—H78	0.9500	B41—B42	1.785 (18)
C79—C84	1.395 (7)	B41—H41A	0.9900
C79—C80	1.398 (8)	B41—H41B	0.9900
C79—P3	1.802 (6)	B41—H45A	1.21 (3)
C80—C81	1.377 (8)	B41—H15G	0.21 (15)
C80—H80	0.9500	B42—B43	1.769 (17)
C81—C82	1.393 (8)	B42—H42A	0.9900
C81—H81	0.9500	B42—H42B	0.9900
C82—C83	1.371 (9)	B42—H45A	1.20 (3)
C82—H82	0.9500	B42—H45B	1.21 (3)
C83—C84	1.378 (8)	B42—H15G	1.59 (11)
C83—H83	0.9500	B43—H43A	0.9900
C84—H84	0.9500	B43—H43B	0.9900
C91—C96	1.395 (7)	B43—H45B	1.21 (3)
C91—C92	1.396 (7)	B43—H15H	1.31 (7)
C91—P4	1.794 (6)	B141—B143	1.758 (16)
C92—C93	1.376 (8)	B141—B142	1.801 (15)
C92—H92	0.9500	B141—H14A	0.9900
C93—C94	1.391 (8)	B141—H14B	0.9900
C93—H93	0.9500	B141—H45B	1.23 (6)
C94—C95	1.393 (8)	B141—H15G	1.18 (3)
C94—H94	0.9500	B142—B143	1.736 (16)
C95—C96	1.382 (8)	B142—H14C	0.9900
C95—H95	0.9500	B142—H14D	0.9900
C96—H96	0.9500	B142—H45A	1.06 (8)
C97—C102	1.391 (7)	B142—H15G	1.20 (3)
C97—C98	1.400 (7)	B142—H15H	1.18 (3)
C97—P4	1.804 (6)	B143—H14E	0.9900
C98—C99	1.380 (8)	B143—H14F	0.9900
C98—H98	0.9500	B143—H45B	0.76 (9)
C99—C100	1.399 (8)	B143—H15H	1.20 (3)
			
C6—C1—C2	120.6 (5)	C121—C122—H122	120.5
C6—C1—P1	122.2 (4)	C123—C122—H122	120.5
C2—C1—P1	117.2 (4)	C124—C123—C122	120.1 (5)
C3—C2—C1	119.2 (5)	C124—C123—H123	120.0
C3—C2—H2	120.4	C122—C123—H123	120.0
C1—C2—H2	120.4	C125—C124—C123	120.7 (5)
C4—C3—C2	119.6 (5)	C125—C124—H124	119.7
C4—C3—H3	120.2	C123—C124—H124	119.7
C2—C3—H3	120.2	C124—C125—C126	119.6 (6)
C5—C4—C3	121.2 (5)	C124—C125—H125	120.2
C5—C4—H4	119.4	C126—C125—H125	120.2
C3—C4—H4	119.4	C121—C126—C125	119.8 (5)
C4—C5—C6	120.3 (5)	C121—C126—H126	120.1
C4—C5—H5	119.9	C125—C126—H126	120.1
C6—C5—H5	119.9	C132—C127—C128	120.3 (4)
C5—C6—C1	119.1 (5)	C132—C127—P5	117.9 (4)
C5—C6—H6	120.4	C128—C127—P5	121.8 (4)
C1—C6—H6	120.4	C129—C128—C127	118.8 (5)
C8—C7—C12	120.1 (4)	C129—C128—H128	120.6
C8—C7—P1	117.8 (4)	C127—C128—H128	120.6
C12—C7—P1	122.0 (4)	C130—C129—C128	120.4 (5)
C9—C8—C7	120.9 (5)	C130—C129—H129	119.8
C9—C8—H8	119.5	C128—C129—H129	119.8
C7—C8—H8	119.5	C129—C130—C131	121.1 (5)
C8—C9—C10	119.3 (5)	C129—C130—H130	119.4
C8—C9—H9	120.3	C131—C130—H130	119.4
C10—C9—H9	120.3	C132—C131—C130	118.8 (5)
C11—C10—C9	119.8 (5)	C132—C131—H131	120.6
C11—C10—H10	120.1	C130—C131—H131	120.6
C9—C10—H10	120.1	C127—C132—C131	120.6 (5)
C10—C11—C12	121.9 (4)	C127—C132—H132	119.7
C10—C11—H11	119.0	C131—C132—H132	119.7
C12—C11—H11	119.0	C134—C133—C138	120.4 (4)
C11—C12—C7	117.9 (5)	C134—C133—P5	121.5 (4)
C11—C12—H12	121.1	C138—C133—P5	118.1 (4)
C7—C12—H12	121.1	C135—C134—C133	118.9 (5)
C14—C13—C18	119.5 (5)	C135—C134—H134	120.5
C14—C13—P1	122.9 (4)	C133—C134—H134	120.5
C18—C13—P1	117.6 (4)	C134—C135—C136	120.9 (5)
C15—C14—C13	120.0 (5)	C134—C135—H135	119.5
C15—C14—H14	120.0	C136—C135—H135	119.5
C13—C14—H14	120.0	C137—C136—C135	120.3 (5)
C14—C15—C16	120.0 (5)	C137—C136—H136	119.9
C14—C15—H15	120.0	C135—C136—H136	119.9
C16—C15—H15	120.0	C136—C137—C138	119.8 (5)
C17—C16—C15	120.1 (6)	C136—C137—H137	120.1
C17—C16—H16	119.9	C138—C137—H137	120.1
C15—C16—H16	119.9	C137—C138—C133	119.5 (5)
C18—C17—C16	120.2 (5)	C137—C138—H138	120.2
C18—C17—H17	119.9	C133—C138—H138	120.2
C16—C17—H17	119.9	C144—C139—C140	119.8 (5)
C17—C18—C13	120.0 (5)	C144—C139—P5	122.7 (4)
C17—C18—H18	120.0	C140—C139—P5	117.4 (4)
C13—C18—H18	120.0	C141—C140—C139	119.0 (4)
C24—C19—C20	120.2 (5)	C141—C140—H140	120.5
C24—C19—P1	122.5 (4)	C139—C140—H140	120.5
C20—C19—P1	117.3 (4)	C140—C141—C142	121.5 (5)
C21—C20—C19	120.3 (5)	C140—C141—H141	119.3
C21—C20—H20	119.8	C142—C141—H141	119.3
C19—C20—H20	119.8	C141—C142—C143	119.0 (5)
C20—C21—C22	119.5 (5)	C141—C142—H142	120.5
C20—C21—H21	120.3	C143—C142—H142	120.5
C22—C21—H21	120.3	C144—C143—C142	120.8 (5)
C23—C22—C21	120.5 (5)	C144—C143—H143	119.6
C23—C22—H22	119.8	C142—C143—H143	119.6
C21—C22—H22	119.8	C143—C144—C139	119.9 (5)
C22—C23—C24	120.5 (5)	C143—C144—H144	120.0
C22—C23—H23	119.8	C139—C144—H144	120.0
C24—C23—H23	119.8	C133—P5—C139	109.5 (2)
C23—C24—C19	119.0 (5)	C133—P5—C127	109.8 (2)
C23—C24—H24	120.5	C139—P5—C127	107.3 (2)
C19—C24—H24	120.5	C133—P5—C121	108.8 (2)
C7—P1—C1	110.5 (2)	C139—P5—C121	110.8 (2)
C7—P1—C13	107.6 (2)	C127—P5—C121	110.7 (2)
C1—P1—C13	110.5 (2)	B3—B1—B2	59.7 (4)
C7—P1—C19	110.1 (2)	B3—B1—H1A	117.8
C1—P1—C19	107.9 (2)	B2—B1—H1A	117.8
C13—P1—C19	110.2 (2)	B3—B1—H1B	117.8
C36—C31—C32	119.9 (5)	B2—B1—H1B	117.8
C36—C31—P2	122.1 (4)	H1A—B1—H1B	114.9
C32—C31—P2	117.9 (4)	B3—B1—H5A	97.9 (15)
C33—C32—C31	119.5 (5)	B2—B1—H5A	42.7 (13)
C33—C32—H32	120.3	H1A—B1—H5A	118.0
C31—C32—H32	120.3	H1B—B1—H5A	84.3
C32—C33—C34	121.4 (5)	B3—B2—B1	59.9 (4)
C32—C33—H33	119.3	B3—B2—H2A	117.8
C34—C33—H33	119.3	B1—B2—H2A	117.8
C35—C34—C33	119.2 (5)	B3—B2—H2B	117.8
C35—C34—H34	120.4	B1—B2—H2B	117.8
C33—C34—H34	120.4	H2A—B2—H2B	114.9
C34—C35—C36	120.4 (5)	B3—B2—H5A	96.7 (15)
C34—C35—H35	119.8	B1—B2—H5A	41.2 (13)
C36—C35—H35	119.8	H2A—B2—H5A	85.4
C31—C36—C35	119.7 (5)	H2B—B2—H5A	118.3
C31—C36—H36	120.1	B3—B2—H5B	41.5 (12)
C35—C36—H36	120.1	B1—B2—H5B	101.3 (14)
C42—C37—C38	119.6 (5)	H2A—B2—H5B	96.4
C42—C37—P2	117.9 (4)	H2B—B2—H5B	103.4
C38—C37—P2	122.4 (4)	H5A—B2—H5B	133 (3)
C39—C38—C37	118.2 (5)	B2—B3—B1	60.4 (4)
C39—C38—H38	120.9	B2—B3—H3A	117.7
C37—C38—H38	120.9	B1—B3—H3A	117.7
C40—C39—C38	122.5 (5)	B2—B3—H3B	117.7
C40—C39—H39	118.8	B1—B3—H3B	117.7
C38—C39—H39	118.8	H3A—B3—H3B	114.9
C39—C40—C41	120.4 (5)	B2—B3—H5B	41.7 (12)
C39—C40—H40	119.8	B1—B3—H5B	101.9 (13)
C41—C40—H40	119.8	H3A—B3—H5B	103.3
C40—C41—C42	118.0 (6)	H3B—B3—H5B	96.2
C40—C41—H41	121.0	B12—B11—B13	60.1 (4)
C42—C41—H41	121.0	B12—B11—H11A	117.8
C37—C42—C41	121.2 (5)	B13—B11—H11A	117.8
C37—C42—H42	119.4	B12—B11—H11B	117.8
C41—C42—H42	119.4	B13—B11—H11B	117.8
C44—C43—C48	119.0 (5)	H11A—B11—H11B	114.9
C44—C43—P2	118.3 (4)	B12—B11—H15A	41.8 (13)
C48—C43—P2	122.6 (4)	B13—B11—H15A	101.8 (14)
C45—C44—C43	120.1 (5)	H11A—B11—H15A	101.4
C45—C44—H44	119.9	H11B—B11—H15A	98.0
C43—C44—H44	119.9	B11—B12—B13	60.2 (4)
C44—C45—C46	120.2 (6)	B11—B12—H12A	117.8
C44—C45—H45	119.9	B13—B12—H12A	117.8
C46—C45—H45	119.9	B11—B12—H12B	117.8
C47—C46—C45	120.1 (5)	B13—B12—H12B	117.8
C47—C46—H46	120.0	H12A—B12—H12B	114.9
C45—C46—H46	120.0	B11—B12—H15A	40.9 (13)
C48—C47—C46	120.1 (5)	B13—B12—H15A	101.0 (14)
C48—C47—H47	120.0	H12A—B12—H15A	98.5
C46—C47—H47	120.0	H12B—B12—H15A	101.8
C47—C48—C43	120.5 (5)	B11—B12—H15B	99.6 (14)
C47—C48—H48	119.7	B13—B12—H15B	40.7 (13)
C43—C48—H48	119.7	H12A—B12—H15B	109.6
C54—C49—C50	118.9 (5)	H12B—B12—H15B	91.9
C54—C49—P2	119.2 (4)	H15A—B12—H15B	140 (2)
C50—C49—P2	121.9 (4)	B12—B13—B11	59.8 (4)
C51—C50—C49	119.9 (5)	B12—B13—H13A	117.8
C51—C50—H50	120.1	B11—B13—H13A	117.8
C49—C50—H50	120.1	B12—B13—H13B	117.8
C50—C51—C52	120.4 (5)	B11—B13—H13B	117.8
C50—C51—H51	119.8	H13A—B13—H13B	114.9
C52—C51—H51	119.8	B12—B13—H15B	41.2 (13)
C53—C52—C51	120.4 (6)	B11—B13—H15B	99.6 (14)
C53—C52—H52	119.8	H13A—B13—H15B	109.5
C51—C52—H52	119.8	H13B—B13—H15B	91.6
C54—C53—C52	119.5 (6)	B23—B21—B22	59.6 (4)
C54—C53—H53	120.2	B23—B21—H21A	117.8
C52—C53—H53	120.2	B22—B21—H21A	117.8
C53—C54—C49	120.8 (5)	B23—B21—H21B	117.8
C53—C54—H54	119.6	B22—B21—H21B	117.8
C49—C54—H54	119.6	H21A—B21—H21B	115.0
C49—P2—C37	108.0 (2)	B23—B21—H25A	101.0 (14)
C49—P2—C43	110.5 (3)	B22—B21—H25A	41.7 (13)
C37—P2—C43	110.8 (2)	H21A—B21—H25A	103.4
C49—P2—C31	109.8 (2)	H21B—B21—H25A	96.4
C37—P2—C31	110.1 (2)	B23—B22—B21	59.9 (4)
C43—P2—C31	107.6 (2)	B23—B22—H22A	117.8
C66—C61—C62	120.8 (5)	B21—B22—H22A	117.8
C66—C61—P3	118.8 (4)	B23—B22—H22B	117.8
C62—C61—P3	120.4 (4)	B21—B22—H22B	117.8
C63—C62—C61	117.9 (5)	H22A—B22—H22B	114.9
C63—C62—H62	121.0	B23—B22—H25A	101.3 (14)
C61—C62—H62	121.0	B21—B22—H25A	41.7 (13)
C64—C63—C62	121.6 (5)	H22A—B22—H25A	96.3
C64—C63—H63	119.2	H22B—B22—H25A	103.4
C62—C63—H63	119.2	B23—B22—H25B	41.7 (13)
C63—C64—C65	120.5 (5)	B21—B22—H25B	99.6 (15)
C63—C64—H64	119.8	H22A—B22—H25B	89.5
C65—C64—H64	119.8	H22B—B22—H25B	111.6
C66—C65—C64	119.1 (5)	H25A—B22—H25B	138 (3)
C66—C65—H65	120.5	B22—B23—B21	60.6 (4)
C64—C65—H65	120.5	B22—B23—H23A	117.7
C65—C66—C61	120.1 (5)	B21—B23—H23A	117.7
C65—C66—H66	120.0	B22—B23—H23B	117.7
C61—C66—H66	120.0	B21—B23—H23B	117.7
C72—C67—C68	119.4 (5)	H23A—B23—H23B	114.8
C72—C67—P3	118.4 (4)	B22—B23—H25B	42.9 (13)
C68—C67—P3	122.2 (4)	B21—B23—H25B	101.5 (15)
C69—C68—C67	118.8 (5)	H23A—B23—H25B	111.0
C69—C68—H68	120.6	H23B—B23—H25B	88.5
C67—C68—H68	120.6	B32—B31—B33	60.4 (4)
C68—C69—C70	121.2 (5)	B32—B31—H31A	117.7
C68—C69—H69	119.4	B33—B31—H31A	117.7
C70—C69—H69	119.4	B32—B31—H31B	117.7
C69—C70—C71	119.8 (6)	B33—B31—H31B	117.7
C69—C70—H70	120.1	H31A—B31—H31B	114.9
C71—C70—H70	120.1	B32—B31—H35A	44.6 (14)
C72—C71—C70	119.8 (5)	B33—B31—H35A	97.7 (17)
C72—C71—H71	120.1	H31A—B31—H35A	122.5
C70—C71—H71	120.1	H31B—B31—H35A	79.8
C71—C72—C67	121.0 (5)	B31—B32—B33	60.2 (4)
C71—C72—H72	119.5	B31—B32—H32A	117.8
C67—C72—H72	119.5	B33—B32—H32A	117.8
C78—C73—C74	119.6 (4)	B31—B32—H32B	117.8
C78—C73—P3	122.4 (4)	B33—B32—H32B	117.8
C74—C73—P3	118.0 (4)	H32A—B32—H32B	114.9
C75—C74—C73	119.8 (5)	B31—B32—H35A	43.8 (13)
C75—C74—H74	120.1	B33—B32—H35A	96.8 (16)
C73—C74—H74	120.1	H32A—B32—H35A	80.5
C74—C75—C76	120.6 (5)	H32B—B32—H35A	122.7
C74—C75—H75	119.7	B31—B32—H35B	101.3 (14)
C76—C75—H75	119.7	B33—B32—H35B	41.2 (12)
C75—C76—C77	119.9 (5)	H32A—B32—H35B	98.6
C75—C76—H76	120.1	H32B—B32—H35B	101.3
C77—C76—H76	120.1	H35A—B32—H35B	132 (3)
C78—C77—C76	119.3 (5)	B31—B33—B32	59.5 (4)
C78—C77—H77	120.3	B31—B33—H33A	117.8
C76—C77—H77	120.3	B32—B33—H33A	117.8
C73—C78—C77	120.8 (4)	B31—B33—H33B	117.8
C73—C78—H78	119.6	B32—B33—H33B	117.8
C77—C78—H78	119.6	H33A—B33—H33B	115.0
C84—C79—C80	119.9 (5)	B31—B33—H35B	102.5 (13)
C84—C79—P3	122.3 (5)	B32—B33—H35B	43.0 (13)
C80—C79—P3	117.8 (4)	H33A—B33—H35B	100.6
C81—C80—C79	119.1 (6)	H33B—B33—H35B	97.8
C81—C80—H80	120.5	B43—B41—B42	59.8 (7)
C79—C80—H80	120.5	B43—B41—H41A	117.8
C80—C81—C82	121.1 (6)	B42—B41—H41A	117.8
C80—C81—H81	119.4	B43—B41—H41B	117.8
C82—C81—H81	119.4	B42—B41—H41B	117.8
C83—C82—C81	119.1 (6)	H41A—B41—H41B	114.9
C83—C82—H82	120.4	B43—B41—H45A	97 (2)
C81—C82—H82	120.4	B42—B41—H45A	41.8 (15)
C82—C83—C84	121.2 (6)	H41A—B41—H45A	119.4
C82—C83—H83	119.4	H41B—B41—H45A	84.2
C84—C83—H83	119.4	B43—B41—H15G	75 (10)
C83—C84—C79	119.6 (6)	B42—B41—H15G	24 (10)
C83—C84—H84	120.2	H41A—B41—H15G	94.6
C79—C84—H84	120.2	H41B—B41—H15G	130.2
C73—P3—C67	111.3 (2)	H45A—B41—H15G	46 (10)
C73—P3—C61	110.3 (2)	B43—B42—B41	59.4 (8)
C67—P3—C61	107.6 (2)	B43—B42—H42A	117.8
C73—P3—C79	107.3 (2)	B41—B42—H42A	117.8
C67—P3—C79	110.2 (3)	B43—B42—H42B	117.8
C61—P3—C79	110.2 (2)	B41—B42—H42B	117.8
C96—C91—C92	120.0 (5)	H42A—B42—H42B	115.0
C96—C91—P4	118.5 (4)	B43—B42—H45A	97 (2)
C92—C91—P4	121.5 (4)	B41—B42—H45A	42.4 (16)
C93—C92—C91	119.9 (5)	H42A—B42—H45A	83.8
C93—C92—H92	120.1	H42B—B42—H45A	119.4
C91—C92—H92	120.1	B43—B42—H45B	43.2 (15)
C92—C93—C94	119.8 (5)	B41—B42—H45B	95 (2)
C92—C93—H93	120.1	H42A—B42—H45B	123.9
C94—C93—H93	120.1	H42B—B42—H45B	80.6
C93—C94—C95	120.9 (6)	H45A—B42—H45B	137 (2)
C93—C94—H94	119.5	B43—B42—H15G	61 (6)
C95—C94—H94	119.5	B41—B42—H15G	3(4)
C96—C95—C94	119.0 (6)	H42A—B42—H15G	119.7
C96—C95—H95	120.5	H42B—B42—H15G	114.8
C94—C95—H95	120.5	H45A—B42—H15G	42 (6)
C95—C96—C91	120.4 (5)	H45B—B42—H15G	96 (6)
C95—C96—H96	119.8	B41—B43—B42	60.8 (8)
C91—C96—H96	119.8	B41—B43—H43A	117.7
C102—C97—C98	120.5 (5)	B42—B43—H43A	117.7
C102—C97—P4	122.0 (4)	B41—B43—H43B	117.7
C98—C97—P4	117.4 (4)	B42—B43—H43B	117.7
C99—C98—C97	119.1 (5)	H43A—B43—H43B	114.8
C99—C98—H98	120.5	B41—B43—H45B	96 (2)
C97—C98—H98	120.5	B42—B43—H45B	42.9 (15)
C98—C99—C100	121.2 (6)	H43A—B43—H45B	123.6
C98—C99—H99	119.4	H43B—B43—H45B	80.7
C100—C99—H99	119.4	B41—B43—H15H	90 (2)
C101—C100—C99	118.7 (6)	B42—B43—H15H	69 (3)
C101—C100—H100	120.6	H43A—B43—H15H	49.3
C99—C100—H100	120.6	H43B—B43—H15H	151.5
C100—C101—C102	121.4 (6)	H45B—B43—H15H	91 (5)
C100—C101—H101	119.3	B143—B141—B142	58.4 (6)
C102—C101—H101	119.3	B143—B141—H14A	117.9
C101—C102—C97	119.1 (5)	B142—B141—H14A	117.9
C101—C102—H102	120.4	B143—B141—H14B	117.9
C97—C102—H102	120.4	B142—B141—H14B	117.9
C108—C103—C104	119.5 (5)	H14A—B141—H14B	115.1
C108—C103—P4	118.2 (4)	B143—B141—H45B	22 (3)
C104—C103—P4	122.3 (4)	B142—B141—H45B	80 (3)
C105—C104—C103	119.6 (5)	H14A—B141—H45B	110.6
C105—C104—H104	120.2	H14B—B141—H45B	109.0
C103—C104—H104	120.2	B143—B141—H15G	99.1 (18)
C104—C105—C106	120.2 (6)	B142—B141—H15G	41.1 (14)
C104—C105—H105	119.9	H14A—B141—H15G	105.7
C106—C105—H105	119.9	H14B—B141—H15G	95.5
C105—C106—C107	120.6 (5)	H45B—B141—H15G	120 (4)
C105—C106—H106	119.7	B143—B142—B141	59.6 (7)
C107—C106—H106	119.7	B143—B142—H14C	117.8
C106—C107—C108	119.2 (5)	B141—B142—H14C	117.8
C106—C107—H107	120.4	B143—B142—H14D	117.8
C108—C107—H107	120.4	B141—B142—H14D	117.8
C103—C108—C107	120.9 (6)	H14C—B142—H14D	114.9
C103—C108—H108	119.6	B143—B142—H45A	102 (3)
C107—C108—H108	119.6	B141—B142—H45A	65 (4)
C114—C109—C110	120.5 (4)	H14C—B142—H45A	135.0
C114—C109—P4	117.8 (4)	H14D—B142—H45A	54.4
C110—C109—P4	121.6 (4)	B143—B142—H15G	99.5 (17)
C111—C110—C109	118.9 (5)	B141—B142—H15G	40.3 (14)
C111—C110—H110	120.5	H14C—B142—H15G	95.7
C109—C110—H110	120.5	H14D—B142—H15G	105.7
C112—C111—C110	120.6 (5)	H45A—B142—H15G	56 (10)
C112—C111—H111	119.7	B143—B142—H15H	43.7 (15)
C110—C111—H111	119.7	B141—B142—H15H	97 (2)
C111—C112—C113	120.9 (5)	H14C—B142—H15H	81.5
C111—C112—H112	119.5	H14D—B142—H15H	121.4
C113—C112—H112	119.5	H45A—B142—H15H	143 (4)
C114—C113—C112	119.1 (5)	H15G—B142—H15H	129 (7)
C114—C113—H113	120.4	B142—B143—B141	62.0 (6)
C112—C113—H113	120.4	B142—B143—H14E	117.6
C109—C114—C113	119.9 (5)	B141—B143—H14E	117.6
C109—C114—H114	120.0	B142—B143—H14F	117.6
C113—C114—H114	120.0	B141—B143—H14F	117.6
C103—P4—C91	108.1 (2)	H14E—B143—H14F	114.7
C103—P4—C109	111.3 (2)	B142—B143—H45B	98 (3)
C91—P4—C109	110.2 (2)	B141—B143—H45B	36 (3)
C103—P4—C97	110.1 (3)	H14E—B143—H45B	103.2
C91—P4—C97	110.2 (3)	H14F—B143—H45B	100.8
C109—P4—C97	106.9 (2)	B142—B143—H15H	42.7 (15)
C122—C121—C126	120.8 (5)	B141—B143—H15H	98 (2)
C122—C121—P5	122.1 (4)	H14E—B143—H15H	120.9
C126—C121—P5	117.0 (4)	H14F—B143—H15H	81.8
C121—C122—C123	119.0 (5)	H45B—B143—H15H	130 (4)
